# A post-PHEIC review of cultural awareness in the COVID-19 response

**DOI:** 10.3389/phrs.2026.1608243

**Published:** 2026-06-24

**Authors:** Kyoo-Man Ha

**Affiliations:** Rabdan Academy, Abu Dhabi, United Arab Emirates

**Keywords:** COVID-19, cultural competence, emergency culture, emergency response, pandemic preparedness

## Abstract

**Objectives:**

The PHEIC status of COVID-19 was officially lifted by the WHO on May 5, 2023. However, many nations continue to grapple with its impacts, particularly those that have not fully embraced the emergency culture. This study aims to explore ways to enhance emergency culture that rose during the COVID-19 response, ultimately contributing to effective pandemic management.

**Methods:**

A systematic literature review, including the PRISMA 2020 flow diagram and checklist, was used to compare cultural unawareness with cultural awareness across six nations: Japan, Sweden, South Africa, the United States, Brazil, and Australia.

**Results:**

To varying degrees, all six nations exhibited cultural unawareness during their COVID-19 responses, which manifested in issues such as personal responsibilities, herd immunity, inequalities, individualism, economic priorities, language barriers, and other factors.

**Conclusion:**

These six nations must adaptively transform their cultural unawareness into cultural awareness while enhancing leadership, communication, cultural competence, emergency preparedness, and international cooperation. This study offers a comprehensive perspective on the emergency culture surrounding the coronavirus pandemic, emphasizing the critical importance of cultural awareness.

## Introduction

### The rationale of study

In various regions of the world, the frequency and intensity of emergencies have not decreased. Thus, all nations must prepare for emergency situations, necessitating an examination of the root causes and underlying drivers of such events [1, 2]. These root causes encompass both structural and non-structural measures and require a paradigm shift following a review of existing knowledge.

Toward the goal of emergency management across different regions—aiming to reduce human losses, economic damages, and psychological impacts—one of the most significant challenges is transforming cultural factors, along with their interrelated sub-factors, from negative to positive. This transformation is crucial, as cultural elements are deeply embedded in emergency management. Nevertheless, individual understanding of emergency culture remains limited [3]. The cultural aspect has often been overlooked in many areas of emergency management [4].

Not everyone reacts to an epidemic in the same manner. Some individuals may exhibit unexpected or even deviant behaviors in response to an outbreak. This raises the question of why reactions to disease outbreaks vary among individuals. Research indicates that individual responses to pandemic emergencies are significantly influenced by cultural components, beyond mere scientific or political factors [5]. Consequently, emergency culture has profoundly shaped the pandemic responses of nations [6].

The World Health Organization (WHO) declared on May 5, 2023, that the outbreak of coronavirus disease 2019 (COVID-19) would no longer be classified as a Public Health Emergency of International Concern (PHEIC) [7]. However, this does not imply that the pandemic is entirely over. The international community continues to grapple with the significance of emergency culture in pandemic management. As part of the emergency culture, individuals have made efforts to foster solidarity during the COVID-19 outbreak by disseminating encouraging messages through online communities, forming cross-cultural organizations, and providing moral support to those infected [8]. Consequently, the enduring impacts of the COVID-19 outbreak have highlighted the need for sustainable cultural models in pandemic management.

Given the above discussion, the present study poses the research question of how effectively the field of COVID-19 emergency response has addressed the issue of emergency culture. This research aims to explore ways to enhance emergency culture during the coronavirus response. The findings will contribute to the overarching goal of pandemic management, which includes mitigating both the physical and social impacts of the COVID-19 outbreak.

Further, the study compares cultural unawareness with cultural awareness across six nations: Japan, Sweden, South Africa, the United States, Brazil, and Australia. A key finding is that these nations must transform their cultural unawareness into cultural awareness to improve their pandemic response. Simultaneously, they need to adequately address issues related to leadership, communication, cultural competence, emergency preparedness, and international cooperation.

### Literature review

According to the Merriam-Webster Dictionary (2026) [9], culture is defined as “a particular society that has its own characteristic features of everyday existence (as pastimes or a way of life).” In emergency situations, individuals often rely on familiar patterns, such as where to obtain appropriate information, where to evacuate, what actions to take, and whom to consult [10]. These cultural patterns develop either intentionally or unintentionally, often without systematic consideration, and significantly influence human behavior across various domains.

The issue of culture is complex due to the numerous interrelated sub-factors involved. For instance, emergency culture is frequently compromised due to the lack of budget allocations. Consequently, professionals such as medical staff, emergency managers, and decision-makers may lack sufficient resources and protection. Therefore, addressing emergency culture requires a comprehensive perspective that considers multiple sub-factors and their interrelations [11].

While it is easy to discuss emergency culture in broad terms, operationalizing the subject through empirical methods poses challenges, as each society possesses its own cultural variables, analyses, emphases, and systems [12]. Localized insights are essential for studying emergency culture within a specific region. Without empathetic engagement with others, it is impossible to gain a meaningful understanding of their culture.

In contrast to safety culture, which primarily focuses on prevention within stable systems, the concept of emergency culture in this study encompasses uncertain and dynamic situations. Emergency culture is situated at the intersection of well-established frameworks in the social sciences and public health, providing a logical foundation for this concept. For example, the Health Belief Model illustrates how individual reactions during emergencies are shaped at the micro level by perceived vulnerability, severity, benefits, and barriers [13]. A macro-level perspective, which examines how societal characteristics influence emergency-related behaviors, is offered by Geert Hofstede’s Cultural Dimensions Theory [14, 15]. By integrating these viewpoints, this study conceptualizes emergency culture as that which connects societal reactions to emergencies, personal health behaviors, and disaster management systems.

Emergency culture is closely aligned with pandemic preparedness, emphasizing the capacities, resources, and coordination systems necessary to anticipate and identify infectious disease outbreaks [16]. Furthermore, emergency culture is closely connected to disaster management, a comprehensive and established field that encompasses the whole disaster management cycle [17]. This study places emergency culture within the current scholarly discourse by clearly linking it to these established frameworks, thereby, enhancing its relevance and accessibility for researchers in public health and disaster management.

Besides, it is essential to collect comparable information and data while applying a cross-cultural perspective. Without such data, the issue of equivalence among comparative objects cannot be adequately addressed. Considering that the nature of emergency culture is idiosyncratic and requires evaluation across various contexts, the cross-cultural perspective is less comparable than other areas, such as curricula, organization, and politics [18]. Further, because the topic of emergency culture continues to evolve during the COVID-19 response, obtaining comparable information and data has become increasingly challenging.

To tackle the issue of non-comparability, the cross-cultural perspective should encompass the whole community within each comparative object [19]. This means that the perspective should be grounded in the various sub-factors present in the community. When attention is given to detailed aspects of a subject, such as emergency culture, comparability may be hindered due to the differing, rather than fixed, cultural values among the units of analysis. For instance, regarding the issue of wearing face masks, many individuals in Asia are more comfortable adopting this behavior as part of their emergency culture, in contrast to those in North America or Europe. Therefore, when comparing the practice wearing of face masks across these continents, the cross-cultural perspective may not be fully applicable due to the differences in associated cultural values.

The issue of emergency culture is integral to human life in a changing environment [20]. Any aspect of emergency management must consider cultural considerations to ensure improved living standards; otherwise, such improvements may not be realized. Emergency culture plays a vital role in emergency management, primarily because it can influence public perception and behavioral changes during an emergency [21, 22]. Consequently, cultural factors significantly shape the issue of emergency interventions, protective measures, and related activities. At the same time, emergency culture affects how individuals and organizations garner public support, particularly through the sharing of values and beliefs.

For example, the extent of risk perception in society does not fluctuate automatically; rather, it is influenced by several complicated factors, including fear, financial considerations, stigma, novelty, and performance. The interplay between risk perception and culture has recently gained recognition in the field of emergency management [23]. Thus, several studies have begun to investigate this topic, with some researchers suggesting that cultural factors are a critical missing link in understanding trends in risk perception across different regions.

During the peak of research on emergency culture in the 1960s and 1970s, American researchers Anderson Moore, Dennis Wenger, and Jack Weller sought to systematically examine the significance of culture in emergencies [24]. Since then, however, there has been a notable decline in rigorous studies on this topic. While some scholars, such as Susanna Hoffman and Anthony Oliver-Smith, along with institutions like the International Federation of Red Cross and Red Crescent Societies, have recently published works on the subject. Nevertheless, a new era of intensive research has yet to emerge.

A review of the existing literature indicates that emergency culture encompasses both strengths and vulnerabilities within the field of emergency management [25]. Consequently, the emergency culture in a specific region can either mitigate or increase the risks associated with emergencies [26]. A positive emergency culture enhances the effective management of emergency impacts, while a negative one does not. Thus, the role of emergency culture is not static.

Thanks to indigenous knowledge, certain local communities in South Asia have developed the capacity to prepare for tsunamis. Thus, the impact of the massive tsunamis on South Asians that struck the Indian Ocean in 2004 was relatively minimal [27]. Similarly, the Simeulueans near Sumatra and the Moken around the Surin Islands have cultivated knowledge on evading tsunami impacts as part of their cultural heritage, successfully passing this wisdom to future generations and significantly reducing human losses.

Some Indonesians strongly believe in volcanic legends rather than scientific data regarding eruptions. Following a volcano erupted in central Java in 2006, many residents evacuated to safe places [28]. However, some returned repeatedly to their homes and farms in the affected areas, primarily to prevent looting. Based on local legends, these individuals assessed the threat of a volcanic eruption as lower than the risk of theft. Hence, some who remained in danger zones ended up losing their lives.

The present research focuses on the emergency response phase during the COVID-19 outbreak. The pandemic management cycle comprises four phases: emergency prevention/mitigation, preparedness, response, and recovery [29]. Although the emergency response phase typically spans a short duration, response to the COVID-19 has extended over a prolonged period. This phase has involved addressing the coronavirus infection through direct measures, including the implementation of various emergency plans and mitigation strategies.

Without exception, emergency culture has played a crucial role in responding to the COVID-19 outbreak, as it provided health, hope, comfort, and inspiration to individuals [30]. An effective cultural response considers the reality of the pandemic, including human losses, economic damages, psychological effects, and inequality issues. Thus, emergency culture can aid in repairing social structures and create new avenues for pandemic management.

To date, few researchers have thoroughly explored the issue of culture in COVID-19 management [31]. Furthermore, those who have addressed the topic have only partially touched on it [32]. Most have relied on a dichotomous approach toward increasing the significance of emergency culture, categorizing it into two dimensions: independent versus interdependent culture or loose versus tight culture. North America and Western Europe are generally classified as having independent or loose cultures, while the rest of the world is often categorized as interdependent or tight cultures.

Accredited researchers have summarized the rationale for systematically incorporating emergency culture into pandemic management, particularly during the COVID-19 outbreak. Three primary reasons have garnered significant support [33]. First, human behavior in pandemic management is incomprehensible without considering culture as a framework. Second, culture reflects the nature of disaster risk reduction. Third, culture can either help or impede residents’ ability to cope with various hazards.

Other reasons have been identified to support the role of emergency culture [34]. Cultural traditions, values, and identities are crucial factors for community resilience, as they can improve disrupted social networks during a pandemic emergency. Furthermore, effective emergency interventions necessitate a high level of trust among stakeholders, making cultural considerations essential in affected areas.

This study offers potential value beyond the existing knowledge base. First, it employs a comprehensive perspective and localized insights to outline the nature of emergency culture, incorporating various cultural sub-factors. Second, unlike previous studies, this research focuses specifically on the emergency response to the COVID-19 outbreak. Ultimately, this work aims to provide unique alternatives or identify root causes regarding the lasting impact of coronavirus infection by thoroughly examining the aspect of emergency culture.

## Methods

**FIGURE 1:**
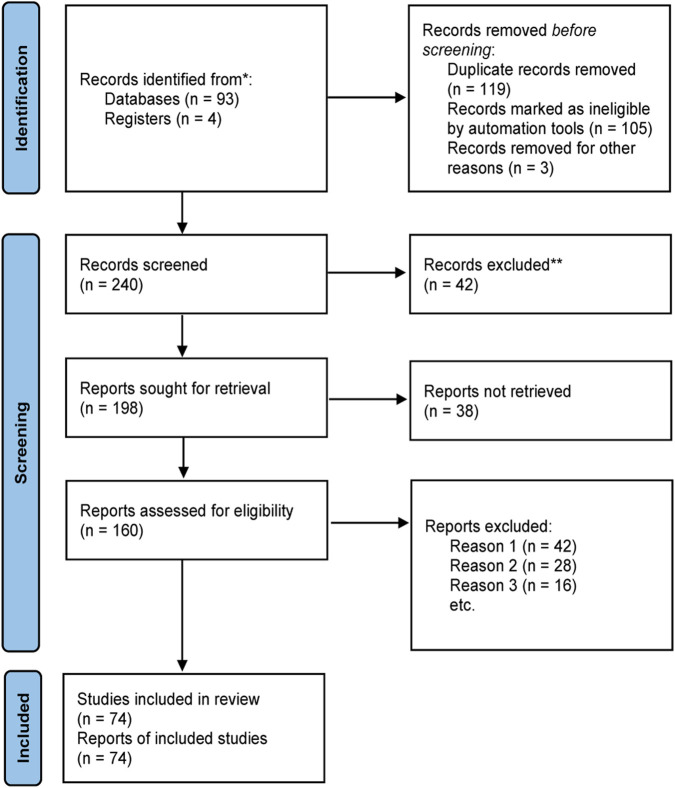
PRISMA 2020 flow diagram [37] (Worldwide, 2026).

To understand the complex human behaviors in various contexts, particularly concerning COVID-19 emergency culture, this study utilized a systematic literature review as its primary methodology. The process involved four steps: planning, data collection, data analysis, and results presentation [35, 36]. As detailed in Table 1, qualitative texts were searched, identified, categorized, interpreted, and recorded. Figure 1 illustrates the application of the Preferred Reporting Items for Systematic Reviews and Meta-Analyses (PRISMA) flow diagram in this study.

**TABLE 1:** Qualitative text inclusion and exclusion (Worldwide, 2026).

Units	Author(s)’s research period for this study
	January 6, 2024 – April 18, 2026
Qualitative texts included	Total of 74 texts (i.e., 37 research articles, 6 books, 17 organizational documents, and 14 key websites)
Criteria for qualitative text inclusion/exclusion	Whether an English text was related to issues of emergency culture, local culture, pandemic management, COVID-19, and national culture
Databases used	Google scholar, EBSCOhost, ScienceDirect, Oxford University Press, MEDLINE/PubMed, Embase, and Scopus among others

Based on the systematic literature review, two major analytical approaches—cultural unawareness and cultural awareness—were identified and contrasted. Cultural unawareness refers to the lack of recognition of both similarities and the differences among individuals and organizations during the COVID-19 emergency response [38]. Cultural awareness, on the other hand, describes the ability of professionals, organizations, and systems to understand, engage with, and respond to individuals from diverse cultural backgrounds [39, 40]. From this perspective, cultural unawareness can be considered as an early or inadequate stage on the continuum of cultural competence. In contrast, cultural awareness signifies a heightened sensitivity to both the similarities and differences among various stakeholders in the emergency response to the COVID-19 outbreak [41]. Thus, cultural unawareness is a detrimental factor, whereas cultural awareness represents a positive alternative in pandemic management.

Six countries were selected as units of analysis for examining the aforementioned two approaches. Since this study does not focus on a specific nation or region but the international community as a whole, the six major continents—Asia, Europe, Africa, North America, South America, and Australia/Oceania—were initially considered as the subjects. Based on a comprehensive web search, six nations, each representing one continent, were identified and included in the study. These countries were Japan, Sweden, South Africa, the United States, Brazil, and Australia [42].

## Results

### Methodological guidelines for major examples of cultural unawareness

In this section, significant measures were taken to enhance methodological rigor and reduce potential bias. A transparent and repeatable protocol was adopted, featuring clearly defined eligibility criteria and adherence to established systematic review guidelines, such as PRISMA, to mitigate the subjectivity inherent in study selection and interpretation [43]. Iterative coding techniques were employed to improve consistency in the absence of inter-rater reliability in qualitative coding. Furthermore, the author critically examined potential biases and meticulously documented analytical conclusions while maintaining reflexivity throughout the study process [44].

### Japan

To hold the Tokyo 2020 Olympic Games, which took place in 2021, on schedule, the Japanese government sought to prove that the country was a safe environment for international athletes, despite the ongoing COVID-19 pandemic. Consequently, Japan focused its coronavirus testing efforts primarily on severe cases rather than conducting widespread testing [45]. Although the International Olympic Committee initially postponed the 2020 Tokyo Olympics, the event ultimately proceeded in 2021, even as the number of confirmed COVID-19 cases in Japan remained relatively high compared with neighboring nations.

In Japanese culture, when a person gets sick, it is that person’s own fault and not other people’s responsibility [46]. Therefore, individuals were expected to take precautions against COVID-19 infection. However, those confirmed to have contracted the virus frequently faced bullying, discrimination, and ostracism, leading many Japanese citizens to hesitate in seeking testing or reporting their travel history to health officials.

### Sweden

All Nordic nations except Sweden implemented lockdown measures during the initial response to the COVID-19 outbreak. The Swedish government strongly advocated for the concept of herd immunity. This approach, however, resulted in fluctuating death rates within the country [47]. Notably, a significant number of senior citizens in care homes succumbed to COVID-19.

There are no clear answers as to why the nation experienced such a terrible situation because numerous factors influenced the nation’s pandemic management. Nevertheless, cultural aspects may have played a significant role [48]. Due to the relatively low population density, social distancing and limited public gatherings were already ingrained in Swedish culture prior to the pandemic. Consequently, the concept of herd immunity was culturally acceptable to many residents.

### South Africa

The COVID-19 outbreak in South Africa progressed more slowly than in many other countries, providing the nation with additional time to prepare. However, it ultimately failed to do so effectively due to a lack of economic resources [49]. Many people assume that the spread of epidemic diseases is inevitable in South Africa, leading to minimal efforts to combat them seriously. Furthermore, despite its geographical location within Africa, the South African government has close ties with South America, which exacerbates issues of inequality during emergencies.

Because of cultural factors, South Africa faced difficulties in implementing preventive measures against COVID-19, such as enforcing stay-at-home orders, social distancing, and prohibiting handshakes [50]. For many individuals, staying at home equates an inability to work and earn a living, resulting in the threat of starvation. Additionally, the communal use of bathrooms and toilets makes it nearly impossible to maintain safe distances. Finally, shaking hands while maintaining eye contact is a traditional practice, and refusing a handshake is often perceived as disrespectful.

### The United States

At the peak of the COVID-19 outbreak, the nation appeared divided. Many medical experts urged the public to adhere to science-based recommendations, while U.S. President Donald Trump leveraged the COVID-19 issue to further his political agenda [51]. Under the Biden administration, Americans gained improved access to coronavirus testing and vaccines, with free testing available even without a prescription. Vaccination and testing were also mandated for certain federal workers [52]. Although concerns about pandemic preparedness and intergovernmental coordination remain, the second Trump administration has initiated coordinated preparation efforts—through federal task forces and updated global health strategies—to mitigate potential infectious disease risks associated with the 2026 FIFA World Cup in North America [53].

The predominant ideology in the United States is individualism, leading many Americans to harbor the cultural trait of believing they can act independently [54]. The Centers for Disease Control and Prevention (CDC) has recommended guidelines such as social distancing, mask-wearing, self-quarantine, and vaccination. However, some Americans have disregarded these guidelines and pushed their own agendas, such as hosting maskless parties and reopening businesses prematurely.

### Brazil

Brazil has a universal healthcare system; however, the government has struggled to manage the impacts of the COVID-19 outbreak effectively [55]. The political ideology of former President Jair Bolsonaro was notably distinct, as he often prioritized economic improvement over pandemic management. He did not hesitate to dismiss cabinet members who opposed his views on preventive measures [56].

Brazil is composed of 26 states and one federal district. Because these states have very different cultures, there is a large extent of heterogeneity across the nation. This cultural diversity can influence the spread of coronavirus. For instance, major cities in the southeastern region have low antibody prevalence, while coastal areas around the Amazon River show high antibody prevalence [57]. Nevertheless, the federal government (including the current president, Luiz Inácio Lula da Silva, has disregarded the cultural differences when coordinating containment measures with the states.

### Australia

The Australian government has undertaken various efforts to implement a comprehensive emergency response to the COVID-19 outbreak. These efforts include establishing fever clinics, testing suspected cases, tracing close contacts of confirmed cases, and imposing travel restrictions [58]. Despite these measures, the risk of coronavirus infection has not significantly diminished. Consequently, Australia has secured a supply of the coronavirus vaccine being jointly developed by AstraZeneca and Oxford University.

Approximately 22% of households in Australia do not speak English or struggle to understand the language, primarily consisting of older residents in Victoria and New South Wales [59]. Despite the language barrier, government information regarding coronavirus infection is often disseminated in English [60]. Consequently, the rates of confirmed cases and fatalities in these multilingual communities have been higher than in English-speaking areas.

## Discussion

### Alternatives toward cultural awareness

The significance of cultural awareness in pandemic management must be acknowledged, particularly given the numerous challenges it has presented across all six continents. This issue should not be overlooked, as emergency culture plays a key part of the COVID-19 response [61]. Emergency culture is intrinsically linked to all facets of hazards, emergencies, disasters, and risks; therefore, the importance of cultural awareness must be adequately addressed, as illustrated in Table 2.

**TABLE 2:** Specific alternatives for cultural awareness in the six nations studied [62–64] (Worldwide, 2026).

Nations	Specific alternatives
① Japan	- Individuals infected by COVID-19 should not face discrimination. Instead, the community should support those infected and aid their recovery through psychological measures
② Sweden	- The government should implement restrictive measures for pandemic management from the onset of the COVID-19 outbreak, without relying on a liberal policy of herd immunity
③ South Africa	- The government should provide disaster relief assistance to individuals staying at home, ensure sanitary facilities for social distancing, and promote alternative greeting to avoid handshaking. Additionally, private organizations, in collaboration with the government, should offer online and offline awareness programs for the public
④ The United States	- The American populace should temporarily shift from an individualistic approach to a communitarian ethos during the emergency response to COVID-19
⑤ Brazil	- The federal government, including both presidents, should systematically consider cultural differences among states when coordinating contentious COVID-19 issues
⑥ Australia	- The government should provide multilingual communities with information about coronavirus not only in English but also in their native languages. Similarly, the nation needs to establish related legal requirements under the principle of policy transparency

There are two contrasting approaches in the field of pandemic management: the scientific approach (also known as risk-oriented management) and the political approach (also known as politics-oriented management) [65]. The scientific approach emphasizes the positive contributions of science, particularly scientific evidence, and the promotion of public interests. In contrast, the political approach highlights the detrimental effects of politics, including political maneuvering and devious methods. In general, there has been a greater demand for the scientific approach than for the political one.

Nonetheless, neither science nor politics alone has proven sufficient to address epidemic outbreaks, as emergency culture continues to create a borderline between the two, especially at the operational level [66]. In essence, emergency culture is a shared element of both science and politics. Thus, the field must develop a new framework for managing pandemic outbreaks that incorporates the concept of emergency culture. This work aims to contribute to the existing literature on the interplay between science and politics in this area of research.

One of the most critical performance measures in this field is how individuals manage pandemic outbreaks by improving their own behaviors [67]. When an emergency occurs, personal safety often takes precedence over all else. Consequently, an effective emergency response at the individual level is urgently required. Each person must recognize the fundamental importance of emergency culture.

Emergency culture can serve as either a negative factor or a positive resource in pandemic management, depending on its application. However, as long as individuals cognitively and practically recognize the significance of transitioning to cultural awareness during emergency responses, emergency culture will be viewed as a reservoir of positive resources [68]. This cultural transition can be an effective tool for helping people navigate challenging situations in complex environments.

A diverse range of leadership styles is essential for fostering cultural awareness. Leadership is inherently linked to pandemic outbreaks, as these two elements complement each other [69]. Effective leadership can mitigate the impacts of a pandemic, while a lack of leadership can exacerbate those impacts. Without strong leadership, achieving cultural awareness becomes significantly more difficult.

Both top-down and the bottom-up leadership approaches are necessary. However, because cultural transitions may not be immediately visible, leaders at the top can provide a clear vision for the field. Conversely, individuals at the grassroots level can also start to work on cultural transitions by applying cutting-edge technologies, such as the Internet, mobile phones, and social networks.

Cultural transition must be accompanied by effective communication [70], which involves crafting appropriate and verified messages regarding pandemic outbreaks and disseminating them to various stakeholders. Without careful planning, development, and implementation of effective communication strategies, it would be challenging for multiple stakeholders to shift from cultural unawareness to cultural awareness. Thus, effective communication is a fundamental aspect of cultural transition.

Through effective communication, the negative consequences of the COVID-19 outbreak, such as loss of life and high infection rates, can be mitigated. Effective communication can also maximize the capacity of stakeholders and foster close partnerships in pandemic management [71]. Similarly, it can promote cooperation among individuals by encouraging discussions of contentious issues. In contrast, ineffective communication may lead to partial failures in cooperation.

Cultural competence refers to the ability of individuals within a region to interact flexibly with those from other regions, particularly by sharing cross-cultural perspectives [72]. Such competence reflects the ability of individuals to function positively at the institutional level, beyond the individual level. Consequently, cultural competence enables institutions to recognize and embrace diverse values and practices related to COVID-19 during emergencies.

Cultural competence cannot be automatically achieved at the national level. Therefore, the six nations examined in this study must develop a foundational understanding of the political, economic, and social contexts surrounding the COVID-19 outbreak. Additionally, they must pursue a nationwide transition to cultural awareness. Each nation should also strive for cultural competence in various aspects of managing the coronavirus infection, including service preparation, policy-making, public administration, and private sector management.

The transition to cultural awareness, while closely related to the emergency response phase in this research, will also enhance the phase of emergency preparedness [73]. Integrating cultural awareness into emergency operation plans will significantly strengthen pandemic preparedness. In this regard, different stakeholders should engage in emergency training and exercises that are supported by cultural awareness.

During the cultural transition, the six countries should promote international cooperation with other nations. Given that pandemics can spread across borders, these nations must collaborate with one another and with other countries to effectively control coronavirus spread. They should cultivate a global mindset while also recognizing cultural barriers and alternatives [74]. Thus, emergency culture can provide hope, comfort, and inspiration to the international community.

### Concluding remarks

To enhance the response to the COVID-19 outbreak, this research examined how the issue of emergency culture could be enhanced in six major countries—Japan, Sweden, South Africa, the United States, Brazil, and Australia—to improve responses to the COVID-19 outbreak. To achieve this goal, we compared two approaches: cultural unawareness and cultural awareness, outlining several barriers and alternatives.

The key finding is that these six nations must transform their current high levels of cultural unawareness into cultural awareness. To this end, each nation needs to address various aspects, such as enhancing societal support, strengthening reliance on restrictive measures, implementing government disaster relief programs, promoting a communitarian ethos, recognizing cultural differences, and addressing multilingual communities. Furthermore, these nations must foster leadership, communication, cultural competence, emergency preparedness, and international cooperation.

This research presents several advantages over previous studies, including a comprehensive perspective, localized insights, the application of multiple cultural sub-factors, a focus on emergency response, and an examination of root causes. However, the most significant advantage of this work is its effort to draw a paradigm shift in addressing coronavirus infections. While the direction of this cultural transition is straightforward, it sends a clear message for the field of pandemic management.

In general, cross-cultural studies often face methodological problems, such as a lack of empirical data, as each nation employs its own numerical criteria, foundational theories, and other parameters. These issues also imposed limitations to this research. Although the study flexibly interpreted various qualitative data as a methodological aspect, a substantial amount of numerical data was excluded from the analysis. Furthermore, this study may be inherently subjective based on individual criteria, as it is a single-author systematic literature review, and formal inter-rater reliability could not be established.

The theoretical frameworks in cross-cultural studies should be grounded in cultural science. In contrast to existing cultural analyses, cultural science reconceptualizes the notion of emergency culture and reinterprets its functions. This research examined cultural structures, the use of culture, and cultural dynamics based on qualitative data—elements that align with cultural science; however, it lacked one crucial aspect due to the absence of empirical data. Nevertheless, this research enhances the socioeconomic process while embodying and addressing cultural science.

From the findings of this study, researchers may identify several topics for further exploration, such as paradigm shifts, the contrast between beneficial and detrimental cultural practices, and the application of emergency culture to other epidemics. The notable strengths and potential limitations of this research may also inform future studies; specifically, researchers could support their own paradigm shifts in emergency culture by referencing empirical data or cultural science. In particular, employing an independent coder in subsequent studies could facilitate the evaluation of inter-rater reliability through the computation of a Cohen’s Kappa ($\kappa$) statistic for validation. Ultimately, the adoption of diverse approaches would contribute to the overarching goal of effective international pandemic management.
